# Synphilin-1 Enhances α-Synuclein Aggregation in Yeast and Contributes to Cellular Stress and Cell Death in a Sir2-Dependent Manner

**DOI:** 10.1371/journal.pone.0013700

**Published:** 2010-10-27

**Authors:** Sabrina Büttner, Charlotte Delay, Vanessa Franssens, Tine Bammens, Doris Ruli, Sandra Zaunschirm, Rita Machado de Oliveira, Tiago Fleming Outeiro, Frank Madeo, Luc Buée, Marie-Christine Galas, Joris Winderickx

**Affiliations:** 1 Institute of Molecular Biosciences, University of Graz, Graz, Austria; 2 Functional Biology, Katholieke Universiteit Leuven, Heverlee, Belgium; 3 Alzheimer & Tauopathies, Inserm U837, Lille, France; 4 Jean-Pierre Aubert Research Centre, Université Lille Nord de France, Lille, France; 5 Faculté de Médecine-Pole Recherche, Université du Droit et de la Santé de Lille, Lille, France; 6 Centre Hospitalier Universitaire, Lille, France; 7 Cell and Molecular Neuroscience Unit, Instituto de Medicina Molecular, Lisboa, Portugal; 8 Instituto de Fisiologia, Universidade de Lisboa, Lisboa, Portugal; National Institutes of Health, United States of America

## Abstract

**Background:**

Parkinson's disease is characterized by the presence of cytoplasmic inclusions, known as Lewy bodies, containing both aggregated α-synuclein and its interaction partner, synphilin-1. While synphilin-1 is known to accelerate inclusion formation by α-synuclein in mammalian cells, its effect on cytotoxicity remains elusive.

**Methodology/Principal Findings:**

We expressed wild-type synphilin-1 or its R621C mutant either alone or in combination with α-synuclein in the yeast *Saccharomyces cerevisiae* and monitored the intracellular localization and inclusion formation of the proteins as well as the repercussions on growth, oxidative stress and cell death. We found that wild-type and mutant synphilin-1 formed inclusions and accelerated inclusion formation by α-synuclein in yeast cells, the latter being correlated to enhanced phosphorylation of serine-129. Synphilin-1 inclusions co-localized with lipid droplets and endomembranes. Consistently, we found that wild-type and mutant synphilin-1 interacts with detergent-resistant membrane domains, known as lipid rafts. The expression of synphilin-1 did not incite a marked growth defect in exponential cultures, which is likely due to the formation of aggresomes and the retrograde transport of inclusions from the daughter cells back to the mother cells. However, when the cultures approached stationary phase and during subsequent ageing of the yeast cells, both wild-type and mutant synphilin-1 reduced survival and triggered apoptotic and necrotic cell death, albeit to a different extent. Most interestingly, synphilin-1 did not trigger cytotoxicity in ageing cells lacking the sirtuin Sir2. This indicates that the expression of synphilin-1 in wild-type cells causes the deregulation of Sir2-dependent processes, such as the maintenance of the autophagic flux in response to nutrient starvation.

**Conclusions/Significance:**

Our findings demonstrate that wild-type and mutant synphilin-1 are lipid raft interacting proteins that form inclusions and accelerate inclusion formation of α-synuclein when expressed in yeast. Synphilin-1 thereby induces cytotoxicity, an effect most pronounced for the wild-type protein and mediated via Sir2-dependent processes.

## Introduction

Parkinson's disease (PD) is the most common neurodegenerative movement disorder affecting about 2% of the population over the age of 65 years. Typical symptoms of PD include muscle rigidity, bradykinesia, postural instability and resting tremors. The neuropathological hallmarks of the disease consist of a progressive degeneration of dopaminergic neurons of the substantia nigra pars compacta and the presence of eosinophilic cytoplasmic inclusions called Lewy bodies (LB). In addition to α-synuclein (α-Syn), which is the major component, many other proteins have been detected in LB, including the α-Syn-interacting protein synphilin-1 [1], [2], [3].

α-Syn is a small presynaptic protein of 140 amino acids. Its cellular function is still unknown but a regulatory role in dopamine neurotransmission and synaptic vesicular recycling has been suggested [3]. Recently, it was proposed that α-Syn is involved in vesicular priming and vesicular membrane fusion [4], possibly by ameliorating complex formation of the plasma membrane and vesicular SNARE proteins [5]. In addition, α-Syn has been reported to perform a chaperone-like activity [5], [6], [7], [8]. α-Syn has the propensity to self-assemble and to form oligomeric protofibrils, which can further mature into different types of fibers and aggregates. Though the exact mechanism that initiates oligomerization and aggregation is still elusive, several studies indicated that the process is dependent on the central hydrophobic domain of α-Syn and initiated by membrane binding through its N-terminal repeat region [1], [9], [10]. In addition, oxidative stress [11] as well as modifications of α-Syn, such as tyrosine nitration [12], phosphorylation [13] or C-terminal truncation [14], have been implicated in the process of oligomerization and aggregation.

Synphilin-1 is another presynaptic protein that was first identified by a yeast two-hybrid screen aiming to recover proteins that associate with α-Syn [15]. The physiological function of synphilin-1 is unknown but since the protein binds synaptic vesicles, it was proposed that synphilin-1 exerts a synaptic function in concert with α-Syn [16]. More recent studies suggested that synphilin-1 could act as a modulator of the ubiquitin-proteasome system [17], [18]. Overexpression of synphilin-1 in cell cultures was shown to promote inclusion formation by α-Syn under conditions of proteasome inhibition [19], [20]. Three studies reported that these inclusions represent aggresomes that can be cleared from the cell by autophagy and therefore should be considered as cytoprotective [21], [22], [23]. Interestingly, the capacity of synphilin-1 to form such inclusions apparently decreased upon the introduction of the R621C substitution, a mutation initially identified in German PD patients but later also found in healthy individuals [24], [25]. Moreover, cells that express this R621C mutant appear more susceptible to staurosporine-induced apoptosis [24]. Recent studies examined the repercussions of synphilin-1 when expressed in mice brains, but the phenotypes reported by different groups are inconsistent. One group showed that synphilin-1 was polyubiquitinated and partially insoluble but found no signs of neurodegeneration [26], while another group demonstrated that the presence of ubiquitin-positive inclusions coincided with cell loss in the cerebellum [27]. The overexpression of synphilin-1 in mouse brain by means of adenoviral vectors was reported to induce inclusion formation and cell death of dopaminergic neurons [28]. Most recently, a double transgenic model was generated combining expression of synphilin-1 and the A53T α-Syn mutant and in this case synphilin-1 was found to attenuate α-Syn-induced neuronal decline [23]. Thus, the influence of synphilin-1 expression on cell viability is presently not clear.

The identification of synphilin-1 as interaction partner of α-Syn through a yeast two-hybrid screening implicates that the conditions enabling such an interaction are preserved in this lower eukaryote. In addition, several studies validated the use of yeast as a powerful model to gain further insights and uncover new clues underlying the pathophysiology of proteins associated with neurodegeneration, including α-Syn [29], [30], [31], [32], [33]. These studies were not only instrumental to decipher structural properties of α-Syn associated with inclusion formation [9], [10] but they also allowed to confine α-Syn-mediated toxicity to the impairment of protein quality control systems and defects in ER-to-Golgi vesicular transport as well as endocytosis [31], [32], [34], [35], [36]. Additionally, transgenic yeast models helped to uncover the crucial role of oxidative stress and mitochondrial (dys)function in the execution of apoptotic and necrotic cell death induced by α-Syn [37], [38]. In this study, we expressed wild-type or mutant synphilin-1 in yeast and compared their capacity to form inclusions and to contribute to cellular stress and cell death in the absence or presence of α-Syn.

## Results

### Wild-type synphilin-1 enhances the α-Syn-induced growth defect in yeast

**Figure 1:**
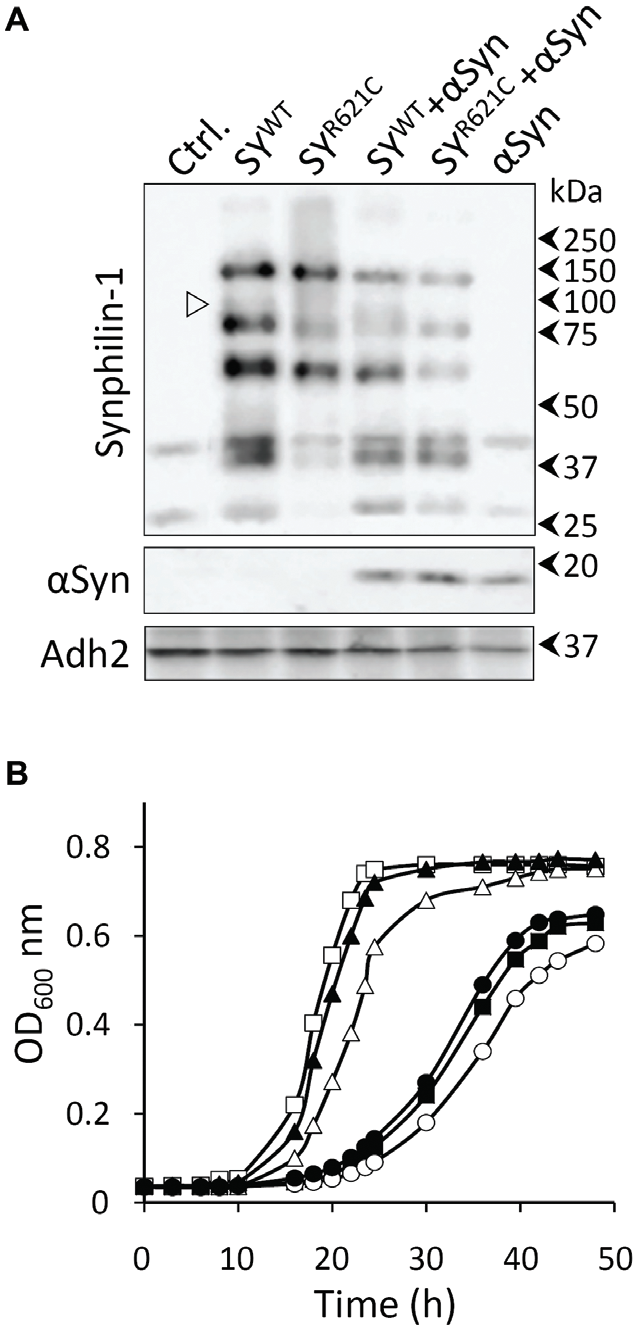
Synphilin-1 and α-Syn toxicity in exponentially growing yeast cells. A: Western blot analysis of control strains transformed with empty plasmids (Ctrl.) or strains expressing SY^WT^ or SY^R621C^ without or with co-expression of α-Syn. The open arrowhead indicates the presence of a 90 kDa proteolytic fragment in case of SY^WT^ expression. Immunodetection was performed using primary antibodies directed against synphilin-1, α-Syn or Adh2 as indicated on the left. Molecular weight markers are indicated on the right. B: Growth curves of the BY4741 wild-type strains transformed with an empty plasmid (□) or constructs allowing for expression of α-Syn (▪), SY^WT^ (Δ), SY^R621C^ (▴),α-Syn and SY^WT^ (○) or α-Syn and SY^R621C^ (•). The data represent the mean of at least three independent transformants.

At present, it is not clear whether synphilin-1 triggers cytotoxicity by itself or solely acts as an accelerator that boosts the cellular stress responses induced by its interaction partner α-Syn. To address this question in more detail, we constitutively expressed wild-type synphilin-1 (SY^WT^), the R621C mutant (SY^R621C^) and α-Syn in *S. cerevisiae*, either alone or in combination, and monitored the effects on growth. The expression was confirmed by Western blot analysis and immunodetection. The proteins SY^WT^ and SY^R621C^ were expressed at similar levels and produced besides a 130 kDa full length protein additional products of 80 kDa, 60 kDa and 40 kDa (Fig. 1A). In addition, SY^WT^ produced a faint 90 kDa band, which was not observed for SY^R621C^. This is consistent with observations previously made upon overexpression of wild-type and mutant synphilin-1 in HEK293 and SH-SY5Y cells [24] as well as in brain tissue from human and transgenic mice [27], [39]. Hence, our data suggest that synphilin-1 is being processed in a similar way in yeast and mammalian cells. As judged from the relative intensities obtained for 80 kDa and 40 kDa products in extracts from cells solely expressing synphilin-1, the efficiency of processing appeared to be different for the wild-type and the mutant protein. However, this difference was less obvious in extracts of cells expressing these proteins in combination with α-Syn. This probably relates to the impairment of protein quality control and clearance mechanisms known to occur upon expression of α-Syn (see [32] and references therein).

Growth analysis demonstrated that the expression of SY^R621C^ in yeast exerted only moderate effects, both when expressed alone or together with α-Syn (Fig. 1B). In contrast, expression of SY^WT^ produced a more pronounced growth phenotype and further aggravated the strong phenotype that we and others previously reported to occur upon expression of α-Syn [30], [31], [40]. To further confirm that these growth defects were not simply due to an overload of the protein expression machinery, we also performed a growth experiment with cells expressing LacZ. As expected, the expression of LacZ did neither produce a growth phenotype, nor did it aggravate the α-Syn-induced growth defect (data not shown).

### Wild-type and mutant synhilin-1 increase the inclusion formation of α-Syn in yeast

**Figure 2:**
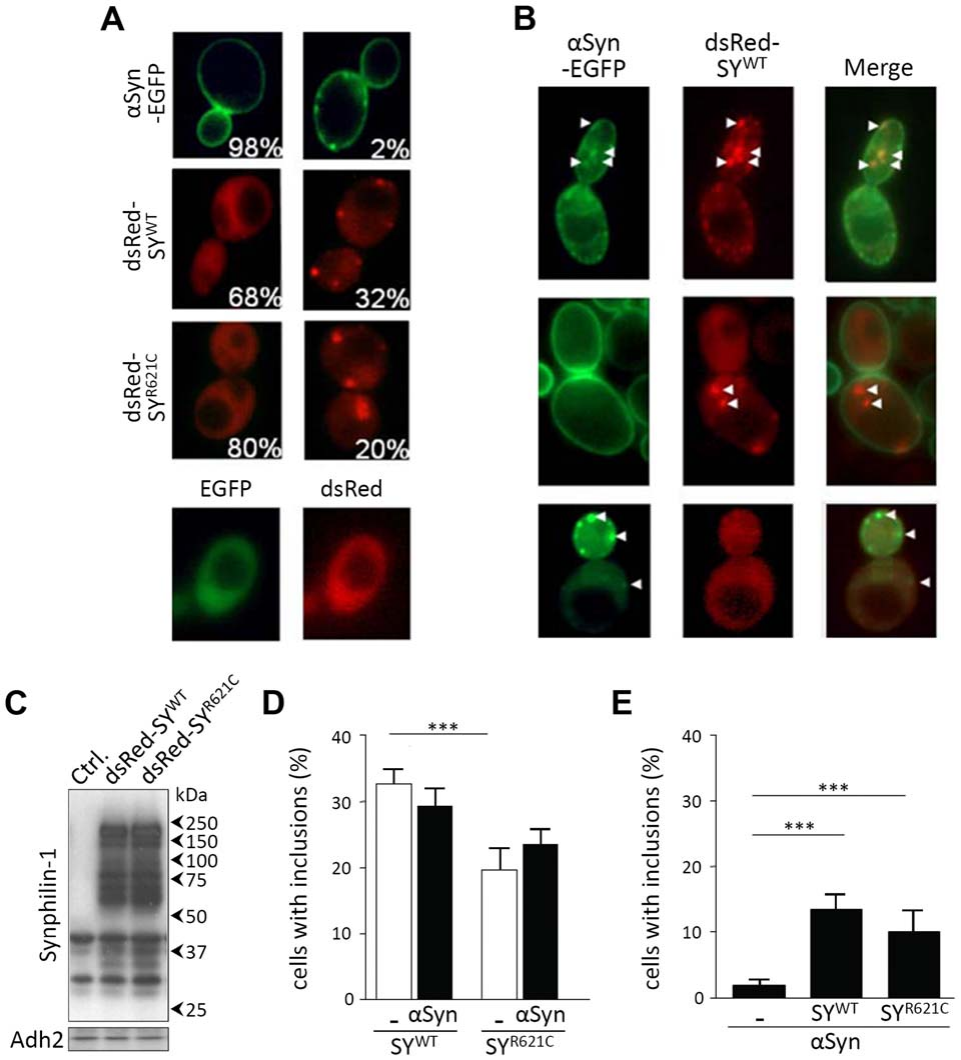
Synphilin-1 induces inclusion formation of α-Syn in yeast. A: Fluorescence microscopic visualization and intracellular localization of the α-Syn–EGFP (upper panels), dsRed-SY^WT^ (middle panels) or dsRed-SY^R621C^ (lower panels) fusion proteins expressed separately in the BY4741 wild-type yeast strain. The panels display cells without (left) or with (right) aggregates. The percentages refer to the number of cells with or without inclusions in an exponential culture. Cells expressing native EGFP or dsRed served as controls and showed a dispersed cytoplasmic localization. B: Fluorescence microscopic visualization and intracellular localization of α-Syn–EGFP and dsRed-SY^WT^ upon combined expression in the BY4741 wild-type yeast strain. The upper panels display cells where both fusion proteins co-localize, the middle panels cells with intracellular inclusions of synphilin-1 and plasma membrane localized α-Syn, and the lower panels cells with peripheral inclusions of α-Syn and a dispersed cytoplasmic distribution of synphilin-1. C: Western blot analysis of strains transformed with an empty plasmid (Ctrl.) or a construct allowing the expression of dsRed-SY^WT^ or dsRed-SY^R621C^. Immunodetection was performed using primary antibodies directed against synphilin-1 or Adh2 as indicated on the left. Molecular weight markers are indicated on the right. D and E: The percentage of cells containing inclusions of wild-type or mutant synphilin-1 (D) or α-Syn (E) in exponential cultures. Data represent the combined results of at least three independent experiments. Error bars represent the variation between different counts. Significance was assayed on the total amount of cells counted using a t-test (*** = p<0.001).

Next, we examined whether the growth properties of the strains expressing SY^WT^ or SY^R621C^ could relate to the capacity of the proteins to form inclusions and to enhance inclusion formation by α-Syn, as previously shown in other cellular and transgenic models [3], [15], [20], [22], [23], [24]. To this end, we expressed α-Syn as C-terminally tagged EGFP fusion and synphilin-1 as N-terminally tagged dsRed fusion and examined the cells in early exponential phase. In line with other studies [30], [31], [40], [41], α-Syn-EGFP localized at the plasma membrane where it started to form inclusions in approximately 2% of the cells (Fig. 2A,E). Both dsRed-SY^WT^ and dsRed-SY^R621C^ displayed a dispersed cytoplasmic distribution in the majority of the yeast cells. However, the proteins concentrated in distinct foci in about one third of the cells expressing dsRed-SY^WT^ and in one fifth of the cells expressing dsRed-SY^R621C^ (Fig. 2A,D). Similar as for α-Syn, these foci appeared to be formed close to the cell periphery in exponential cells. As will be discussed below, the foci transformed into larger inclusions when the cells approached the diauxic shift to enter stationary phase. The difference in the capacity of dsRed-SY^WT^ and dsRed-SY^R621C^ to form foci and inclusions cannot be ascribed to differences in expression, since similar levels were detected upon western blot analysis (Fig. 2C). Hence, our data therefore suggest that the R621C mutation decreases the capacity of the protein to form inclusions, which is consistent with data obtained in SH-SY5Y cells [24]. As described below, inclusions were also evident with similar percentages when SY^WT^ was expressed as EGFP fusion, confirming that inclusion formation is a property associated to synphilin-1 itself and not artificially produced by the fluorescent tag.

Analysis of the strains co-expressing tagged α-Syn and synphilin-1 versions revealed that most of the cells were still inclusion-negative and displayed α-Syn-EGFP at the plasma membrane and synphilin-1 dispersed throughout the cytoplasm. Those cells that formed inclusions fell into three categories (Fig. 2B), with the largest group being the cells harboring synphilin-1 inclusions but no visible α-Syn inclusions. Some of the cells presented inclusions for both proteins, which in many, but not all of the cases, consisted of inclusions where α-Syn-EGFP clearly co-localized with dsRed-SY^WT^ or dsRed-SY^R621C^. In a minor fraction of the cells, α-Syn inclusions were detectable without apparent synphilin-1 inclusions. When quantified, it became clear that α-Syn had only a minor impact on inclusion formation of synphilin-1, as the amount of cells with dsRed-SY^WT^ or dsRed-SY^R621C^ inclusions did not change dramatically (Fig. 2D). Conversely, however, the presence of wild-type or mutant synphilin-1 clearly accelerated inclusion formation by α-Syn. Indeed, the number of cells with α-Syn-EGFP inclusions increased from 2%, in case of expression of α-Syn alone, to 13% in case of combined expression with SY^WT^ and 10% when co-expressed with SY^R621C^ (Fig. 2E). These data favor an unilateral interaction between the proteins in that α-Syn cannot seed inclusion formation by synphilin-1, while synphilin-1 can still recruit α-Syn and thereby propagate nucleation of the latter. Moreover, the data obviously indicate that the level of inclusions formed by these proteins does not correlate with the severity of their associated growth defects when expressed in yeast.

### Synphilin-1 is a lipid raft binding protein

**Figure 3:**
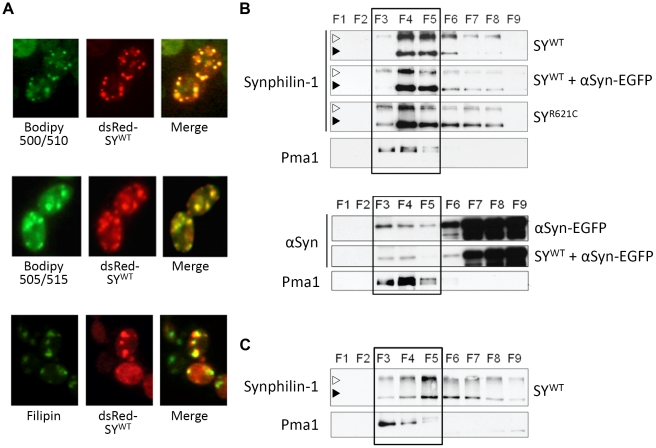
Synphilin-1 interaction with lipid rafts in yeast membranes. A: Co-localization of inclusions formed by dsRed-SY^WT^ with lipid particles and endomembranes (Bodipy 500/510; upper panels), lipid droplets (Bodipy 505/515; middle panels), or sterol-enriched membrane domains (Filipin; lower panels) in yeast. B and C: Interaction with lipid rafts of SY^WT^, SY^R621C^ or α-Syn–EGFP in yeast membranes of the wild-type BY4741 strain (B) or its isogenic *erg24Δ* mutant (C). The proteins expressed are indicated on the right, the antibodies used for immunodetection on the left. Lipid raft fractions (boxed areas) were identified using the yeast lipid raft marker Pma1. The open arrowhead indicates the position of the 250 kDa molecular weight marker, the black arrowhead that of the 150 kDa marker.

Synphilin-1 was shown to bind synaptic vesicles [16] and is known to bind phospholipids, membranes and lipid droplets [39], [42]. Since the membrane-binding properties of α-Syn are thought to be relevant for its pathologic activity [1], [9], [10], we speculated that also for synphilin-1 the binding to different types of lipid structures could be linked to its inclusion formation. To elaborate on this, we incubated exponential cells expressing dsRed-SY^WT^ with the green fluorescent probe C1-BODIPY 500/510-C12, a fatty acid that serves as precursor for the biosynthesis of phospholipids. After one hour incubation, the probe stained distinct small foci, which likely represent sites of newly formed endomembranes. As shown in the overlay, most of these foci co-localized with the inclusions formed by dsRed-SY^WT^ (Fig. 3A, upper panel). Next, we stained cells for lipid droplets with the non-polar green fluorescent probe BODIPY 505/515. Again, co-localization with inclusions formed by dsRed-SY^WT^ could be detected, though we observed that cells contained more inclusions than lipid droplets (Fig. 3A, middle panel). Combined, these data confirm that synphilin-1 is a lipid binding protein and suggest that lipid binding is required for inclusion formation. Finally, to assess whether the inclusion formation of synphilin-1 could involve an interaction with so-called detergent resistant membrane domains (DRMs), also known as lipid rafts, we performed a staining with filipin, a dye specific for sterols [43]. As shown, many of the inclusions formed by dsRed-SY^WT^ in exponential cells localized at sites enriched for sterols (Fig. 3A, lower panel).

To further analyze the capacity of synphilin-1 to bind lipid rafts, we performed fractionations and separated the insoluble membranes by flotation on an Optiprep gradient. For comparison, we included α-Syn-EGFP in this analysis as we previously found that only a minor fraction of native α-Syn and its fusion protein could interact with DRMs [31]. We expected a better interaction of synphilin-1 with DRMs given its higher propensity to form inclusions. Indeed, SY^WT^ was almost entirely recovered in those fractions containing the yeast DRM marker Pma1 (Fig. 3B, upper panel). These fractions not only contained the monomeric 130 kDa form but also a higher-order complex of just more than 250 kDa, which most likely corresponds to a dimeric form. In fact, a very recent study confirmed that synphilin-1 forms antiparallel dimers through its central coiled-coil domain [20]. In contrast to synphilin-1, the fusion protein α-Syn-EGFP was mainly recovered in the fractions containing detergent soluble material (Fig. 3B, lower panel), thereby confirming our previously reported observations. We also investigated whether synphilin-1 and α-Syn mutually affected each other's DRM-binding properties. Although we noticed somewhat reduced dimerization of SY^WT^, no significant changes were observable for lipid raft binding of the monomeric forms upon combined expression. We also did not observe significant changes for the lipid raft binding of α-Syn-EGFP upon co-expression of synphilin-1, but rather a general decrease in α-Syn content in all fractions. Analysis of SY^R621C^ confirmed that this mutant displays altered lipid binding affinities since higher concentrations of the monomeric form were present in the more detergent-soluble fractions. However, the dimeric form of the mutant remained most abundant in the DRM fractions, suggesting that binding to lipid rafts and dimerization could be associated.

In yeast, lipid rafts are enriched in sphingolipids and ergosterol, the yeast counterpart of mammalian cholesterol. To obtain additional confirmation that synphilin-1 is a true lipid raft binding protein, we expressed SY^WT^ in the strain lacking Erg24. *ERG24* encodes the sterol C-14 reductase, which is required for proper ergosterol biosynthesis [44]. Fractionation of membranes of the *erg24Δ* mutant demonstrated that the monomeric form of SY^WT^ was displaced to the more dense fractions containing detergent-soluble material, indicative that synphilin-1 is, indeed a bona fide lipid raft binding protein (Fig. 3C). Also in this case, most dimers of SY^WT^ were still recovered in the DRM fractions ruling out the possibility that dimerization would simply be an artifact of concentrating the monomeric protein in the DRM fractions.

**Figure 4:**
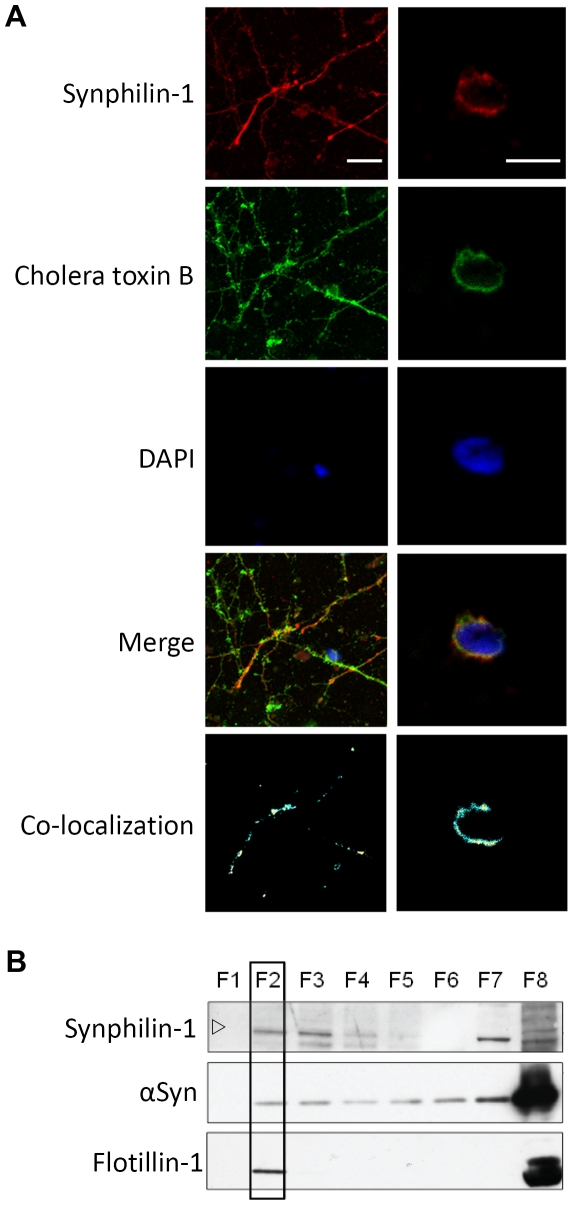
Synphilin-1 interaction with lipid rafts in membranes of mouse primary neurons. A: Pictures obtained by confocal microscopy to determine co-localization of inclusions formed by mouse synphilin-1 with lipid rafts in membranes of mouse primary neuronal cultures. Shown are representative pictures taken at the plane of the neurites (left panels) or of the neuronal cell body (right panels). Synphilin-1 was detected using a goat anti-synphilin 1 as primary antibody and donkey anti-goat IgG (H+L) coupled to Alexa Fluor 568 as secondary antibody. The cholera toxin subunit B served as a marker for lipid rafts. DAPI was used to stain nuclei. The white bar in the upper panel corresponds to a size of 10 µm. B: Interaction with lipid rafts of endogenous SY^WT^ and α-Syn in membranes of mouse primary neuronal cultures. Lipid raft fractions (boxed areas) were identified using the mammalian lipid raft marker flotillin-1. The open arrowhead indicates the position of the 100 kDa molecular weight marker.

To our knowledge, this is the first report of synphilin-1 being a lipid raft binding protein. Therefore, we wondered if the DRM localization of synphilin-1 could be extended to mammalian cells and evaluated the presence of endogenous synphilin-1 in lipid rafts of primary neuronal cultures from the mouse cortex based on co-localization with the cholera toxin B subunit [45]. Confocal fluorescence microcopy confirmed significant co-localization of synphilin-1 and cholera toxin B, both in the neurites (Fig. 4A, left panels) as well as the neuronal cell body (Fig. 4A, right panels). Next, we analyzed the interaction of synphilin-1 with DRMs by means of membrane fractionations. Consistent with previously reported data [16], endogenous synphilin-1 was expressed as a 90 kDa protein in this cellular system and as shown, a substantial amount of the protein was recovered in the same fractions as the mammalian DRM marker flotillin-1 (Fig. 4B). Hence, synphilin-1 is also a lipid raft binding protein in neuronal cells. Note that there is no evidence that mouse synphilin-1 readily forms inclusions in neurons [23], [27] and perhaps this may explain why we only detected the monomeric form and no dimers. To conclude this analysis, we performed immunodetection for endogenous mice α-Syn and found this protein to be mainly distributed over the fractions containing detergent-soluble material, thereby confirming the data obtained in yeast.

### Co-expression of synphilin-1 increases S129-phosphorylation of α-Syn

**Figure 5:**
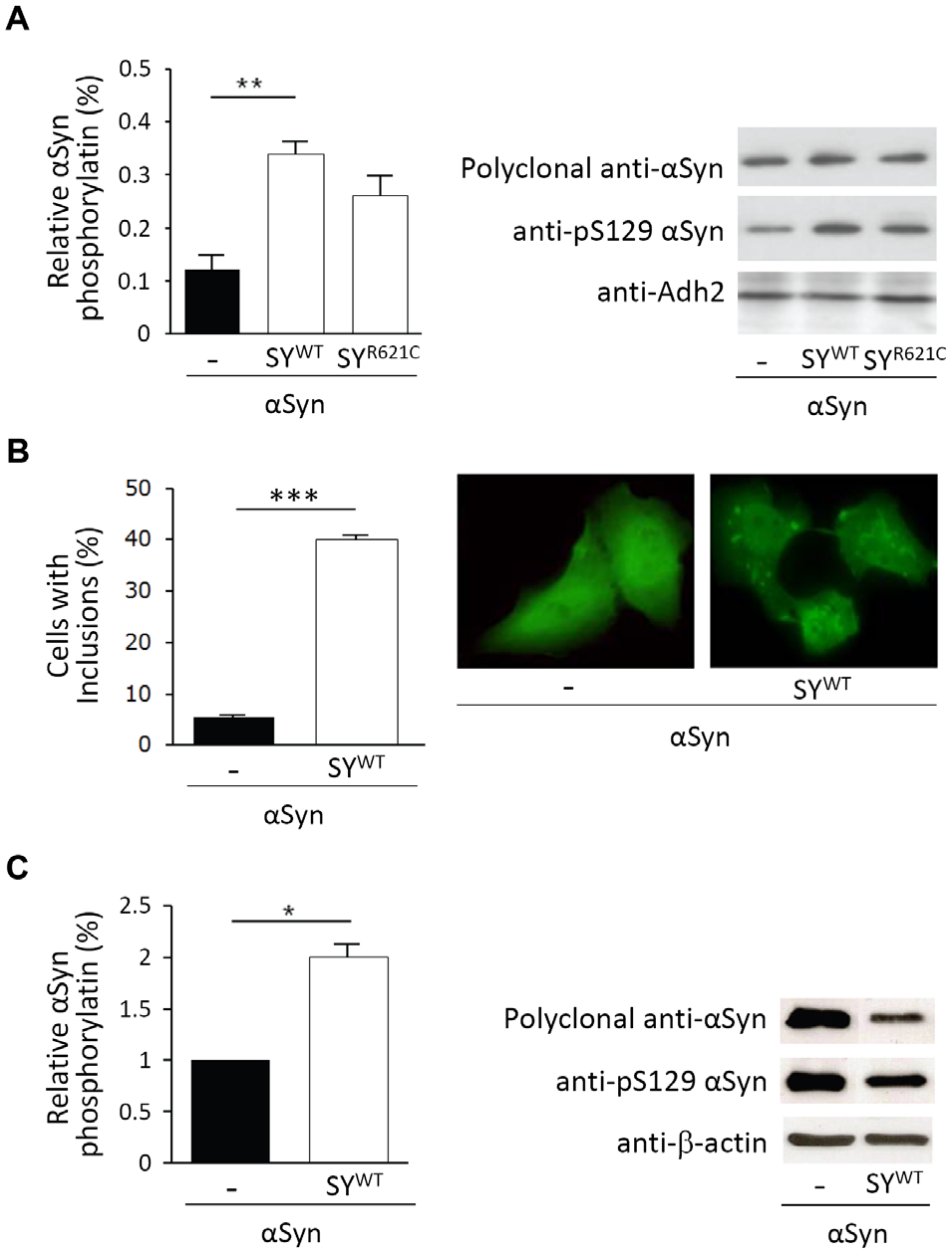
Co-expression of synphilin-1 increases α-Syn S129-phosphorylation. A: Phosphorylation of α-Syn at S129 in the BY4741 wild-type strain when the expression of α-Syn was combined with an empty plasmid or constructs allowing for co-expression of SY^WT^ or SY^R621C^ as indicated. The panel on the left represents the average S129-phosphorylation as determined by immunodetection using a P-S129 specific monoclonal antibody, shown in the right panel, and quantified relative to intensity obtained for immunodetection with a polyclonal α-Syn antibody. B: The left panel shows the average number of H4 neuroglioma cells containing inclusions formed by α-Syn–EGFP when expressed alone or in combination with SY^WT^ as determined by fluorescence microscopic visualization, for which a representative picture is shown in the right panel. C: Phosphorylation of α-Syn at S129 in H4 neuroglioma cells as detected by immunodetection using a P-S129 specific monoclonal antibody and quantified relative to the intensity obtained for immunodetection with a polyclonal α-Syn antibody. The panel on the left represents the relative average phosphorylation, the panel on the right a corresponding Western blot analysis. All data represent the mean ± SEM of at least three independent experiments. Significance was assayed using a 1-way ANOVA (A) or t-test (B and C)(* = p<0.05; ** = p<0.01; *** = p<0.001).

Several studies in brain and transgenic models indicated that α-Syn phosphorylation at serine-129 (S129) is an early event in the pathology of Parkinson's disease [13], [46], [47], [48]. Also in yeast, recent studies confirmed a good correlation between the phosphorylation of α-Syn at S129 and α-Syn-mediated cytotoxicity [31]. Here, we asked whether α-Syn phosphorylation would be increased under conditions where its inclusion formation capacity and cytotoxicity is enhanced by co-expression with synphilin-1. Using a phospho-specific antibody against S129, we compared the immunoreactivity of lysates from cells expressing α-Syn either alone or in combination with SY^WT^ or SY^R621C^. Phosphorylation of α-Syn on S129 was indeed increased both in the presence of wild-type and mutant synphilin-1 (Fig. 5A), but only in case of the former it was significant.

To extend our observation to a mammalian system, we monitored the formation of inclusions and S129-phosphorylation of α-Syn-EGFP when expressed either alone or in combination with SY^WT^ in H4 neuroglioma cells. In line with data obtained with other cell lines [15], [20], only a small number of cells presented inclusions when the fusion protein was overexpressed alone, but this number increased almost eight times upon co-expression with synphilin-1 (Fig. 5B). Similar as in yeast, the enhanced formation of α-Syn-inclusions was paralleled by an increase in S129-phosphorylation (Fig. 5C).

### Synphilin-1 confers toxicity in aged yeast cells

Though many neurodegenerative disorders are tightly associated with ageing, the relationship between synphilin-1-mediated toxicity, ageing and cell death has not been studied in detail. Recently, we performed yeast chronological ageing experiments, a well established model to study oxidative damage and ageing in post-mitotic cells [49], [50], and demonstrated that α-Syn-mediated toxicity correlated with enhanced levels of reactive oxygen species (ROS) produced in the respiratory chain in mitochondria and, as such, with signs of apoptotic and necrotic cell death [32], [37]. As synphilin-1 was shown to render SH-SY5Y cells more sensitive to apoptosis [24], we set out to compare the respective contributions of α-Syn and synphilin-1 to cell death in yeast.

**Figure 6:**
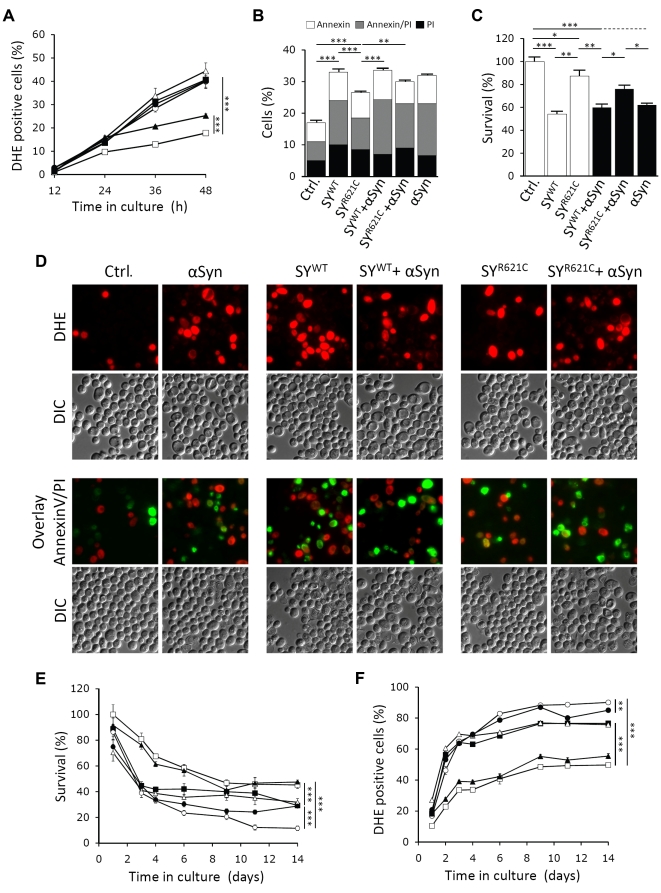
α-Syn and synphilin-1 equally enhance cell death in aged yeast cells. A: Quantification of ROS accumulation using DHE staining at different times during growth of yeast strains transformed with an empty plasmid (□, Ctrl.) or constructs allowing for expression of α-Syn (▪), SY^WT^ (Δ), SY^R621C^ (▴),α-Syn and SY^WT^ (○) or α-Syn and SY^R621C^ (•). B: Quantification of the number of cells that display phosphatidylserine externalization or loss of membrane integrity using annexinV/propidium iodide (PI) co-staining at 36 h of growth in the strains used in A. C: Quantification of viable cells present in the strains used in A at 36 h of growth as determined by their ability to form colonies. D: Fluorescence microscopic visualization of cells expressing combinations of α-Syn, SY^WT^ or SY^R621C^ as indicated and stained with DHE (upper panels) or co-stained with annexinV and PI (lower panels) after 36 h of growth. E and F: Quantification of viable cells (E) and cells producing ROS (F) in the strains used in A when kept in culture for two weeks. All data represent mean ± SEM of six independent transformants. Significance of the data was determined by t-tests (* = p<0.05; ** = p<0.01; *** = p<0.001).

First, we analyzed the effect of α-Syn, SY^WT^ or SY^R621C^ expression on the ROS production at different time points after the inoculation of the cells on fermentative glucose-containing medium. Quantification of ROS production was analyzed by the conversion of non-fluorescent DHE into fluorescent ethidium, a reaction catalyzed by superoxides. As shown, expression of SY^WT^ alone or in combination with α-Syn triggered a significant and equivalent increase in the number of DHE positive cells as compared to the control. Such a marked increase was not observed with the strain expressing only SY^R621C^, mainly because this strain, as well as the control strain, produced less ROS once they traversed the diauxic shift and switched to respiratory growth (Fig. 6A). This growth phase, was only reached by the other strains approximately 12 hours later (see Fig. 1B). To monitor the effect of synphilin-1 and α-Syn expression on cell death and cell survival, we therefore analyzed in more detail a sample taken 36 h after inoculation. As expected, the levels of ROS producing cells correlated nicely with the number of cells displaying signs of apoptotic and necrotic cell death as measured by annexinV and propidium iodide staining, respectively (Fig. 6B,D). The total number of dying cells was equally high for the strains expressing SY^WT^ or α-Syn and lower for strains expressing SY^R621C^, again indicating that the R621C mutation reduced synphilin-1 cytotoxicity. The latter became even more significant when counting the number of surviving cells by means of colony formation. In fact, here we noticed that mutant synphilin-1 somehow protected cells from α-Syn-instigated toxicity as we observed an increase in surviving cells of 16% upon combined expression of α-Syn and SY^R621C^ (Fig. 6C). Such a protective role was not observed for the wild-type protein since the percentages of viable cells were equally low for strains expressing α-Syn with or without co-expression of SY^WT^.

The finding that SY^WT^ and α-Syn induced cell death to a similar extent in cultures approaching stationary phase was rather surprising as it contrasted with the observations made during exponential growth where α-Syn had a more pronounced impact (Fig. 1B). It suggested that the expression of synphilin-1 conferred toxicity mainly when cells were ceasing division, while the expression of α-Syn imposed restrictions at different stages during growth. To investigate this in more detail, we performed a chronological ageing experiment and monitored cell survival (Fig. 6E) and ROS formation (Fig. 6F) when the cultures were kept for a period of two weeks. This confirmed that SY^WT^ and α-Syn equally reduced the chronological life span of aged yeast cells and, interestingly, this effect appeared to be synergistic when the expression of both proteins was combined. As expected, the SY^R621C^ mutant hardly affected chronological life span by itself, but when its expression was combined with α-Syn, we noticed that with time also this mutant protein triggered a synergistic effect as evidenced by enhanced production of ROS and decreased cell viability in comparison to the strain expressing only α-Syn. However, consistent with the data described above, mutant synphilin-1 somehow maintained its protective properties throughout the ageing experiment. Indeed, while the strain that expressed both SY^WT^ and α-Syn showed a continuous decline in cell viability, the strain with combined expression of SY^R621C^ and α-Syn maintained cell viability from the fourth day onward. Hence, this resulted in a progressive increasing difference in the percentage of surviving cells between both strains (p<0.05 at day 6; p<0.01 at day 11;p<0.001 at day 14).

In all experiments described above, we again used cells that expressed LacZ without or with co-expression of α-Syn as additional control. As expected, the expression of LacZ did not trigger toxicity or changes in cell death related phenotypes (data not shown).

### The sirtuin Sir2 mediates synphilin-1 toxicity

**Figure 7:**
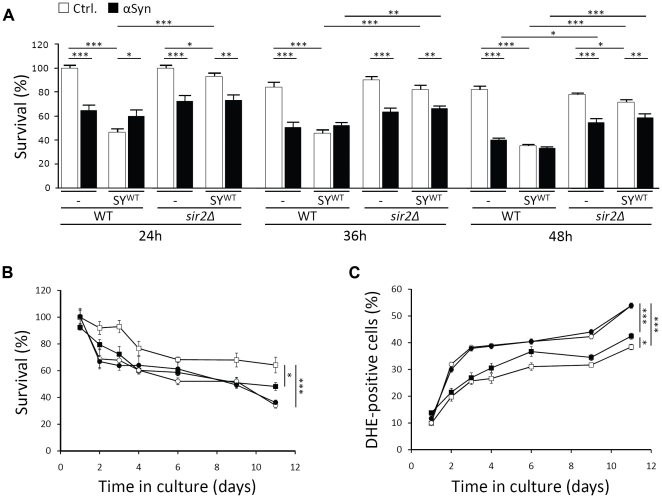
Sir2 mediates synphilin-1 toxicity in yeast. A: Relative quantification of viable cells as determined by their ability to form colonies at different times after inoculation of the wild-type strain or the isogenic *sir2Δ* mutant transformed with empty plasmids or constructs allowing for expression of α-Syn or SY^WT^, either alone or in combination as indicated. The number of viable cells in samples taken after 24 h of growth of the two strains transformed with the empty plasmids was set at 100%. B and C: Quantification of viable cells (B) and cells producing ROS (C) during chronological ageing of the wild-type strain transformed with an empty plasmid (□) or expressing SY^WT^ (▪) and the isogenic *sir2Δ* mutant transformed with an empty plasmid (○) or expressing SY^WT^ (•). All data represent mean ± SEM of six independent transformants. Significance of the data was determined by t-tests (* = p<0.05; ** = p<0.01; *** = p<0.001).

Several age-related phenomena in higher and lower eukaryotes are modulated by the activities of sirtuins or silent information regulators. The yeast sirtuin Sir2 was shown to play both pro- and anti-ageing roles. On the one hand, enhanced Sir2 activity extends replicative longevity or the number of buddings of a mother cell [51], while on the other hand, the protein seems to act as inhibitor of chronological ageing and the ability of yeast cells to maintain their viability in stationary phase [52]. In higher eukaryotes, most studies focussed on the role of SIRT1 as mediator to extend longevity by calorie restriction and on the potential benefits of this protein to prevent neuronal loss [53]. Recent studies, however, reported that inhibition of another member of the sirtuin family, i.e. SIRT2, rescued H4 neuroglioma cells from α-Syn-induced cytotoxicity and prevented dopaminergic cell death in a *Drosophila* model [54], [55]. This observation led us to investigate the possible involvement of Sir2 in mediating synphilin-1 toxicity in our yeast model. First, we analyzed the effect of *SIR2* deletion on the survival of cells expressing SY^WT^ with or without co-expression of α-Syn. Consistent with the observations made in mammalian cells and *Drosophila*, the level of SY^WT^-induced toxicity was significantly lower in the strain lacking Sir2 as compared to the wild-type strain (Fig. 7A). Also α-Syn instigated a lower toxicity in the *sir2Δ* deletion mutant in comparison to the wild-type strain, a difference that became significant once the cells entered stationary phase. Next, we performed a chronological ageing experiment on an independent set of transformants, which carried either a single control plasmid or a plasmid allowing for SY^WT^ expression. As compared to the double transformed cells described above, the synphilin-1-induced toxicity levels were lower in these single transformants. Nevertheless, the difference in survival and ROS production between wild-type cells with or without SY^WT^ expression was still highly significant throughout the course of the experiment (Fig. 7B,C). For the *sir2Δ* strain harboring the control plasmid, we noticed inherent lower survival rates and higher levels of ROS during the ageing experiment. Most interestingly, we could not observe any additional toxic effect induced by synphilin-1 in this mutant strain. Indeed, with the *sir2Δ* strain the curves depicting the number of viable cells or DHE-positive cells, with or without expression of SY^WT^, almost completely overlapped. Hence, it can be concluded that Sir2 plays an essential role in mediating synphilin-1 toxicity.

### Aggresome formation and mitotic partitioning of synphilin-1 inclusions

**Figure 8:**
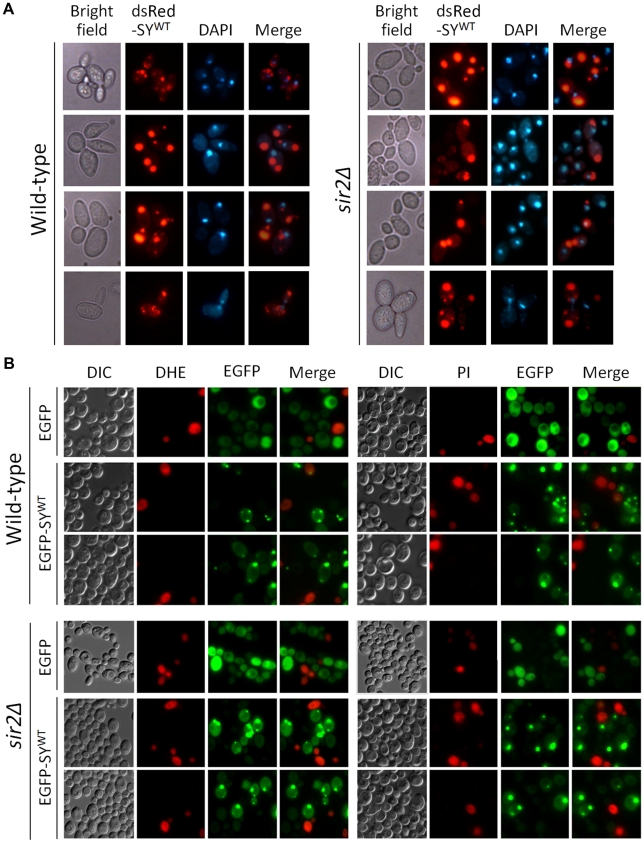
Synphilin-1 forms aggresomes in cells approaching stationary phase. A: Fluorescence microscopy pictures of post-diauxic wild-type and *sir2Δ* cells with large inclusions formed by dsRed-SY^WT^ and stained with DAPI to visualize the nucleus. B: Fluorescence microscopy pictures of post-diauxic wild-type and *sir2Δ* cells expressing either EGFP or EGFP-SY^WT^, as indicated, and stained with DHE to visualize ROS producing cells (left panels) or with PI to discriminate death cells (right panels).

Recent studies in mammalian cells and transgenic mice suggested that upon failure of the proteasome, cells start to accumulate synphilin-1 in aggresomes, which are believed to be cytoprotective since they can be cleared from the cell by autophagy [21], [22], [23]. Aggresomes are formed by convergence of smaller inclusions at the centrosomes via microtubule-based transport. In yeast, the aggresome co-localizes with the spindle pole body [56], the prototype of the centrosome located in the nuclear envelope [57]. As mentioned above, we observed that when the cultures approached the diauxic shift to enter stationary phase many cells displayed one to two large synphilin-1 inclusions. These cells were often also larger in size. To determine whether these inclusions could correspond to aggresomes, we first performed a nuclear staining with DAPI on wild-type and *sir2Δ* cells expressing dsRed-SY^WT^ after 36 h of growth. This revealed that the large inclusion, or one of the inclusions when there were more, was in close proximity of the nucleus in the majority of wild-type and *sir2Δ* cells (Fig. 8A). Also in the strains that combined expression of dsRed-SY^WT^ and α-Syn this appeared to be the case (data not shown). Next, we tested whether the large inclusions were cytoprotective. To this end, we transformed the wild-type and *sir2Δ* strains with a construct allowing expression of a N-terminally tagged EGFP-SY^WT^ fusion. This fusion protein triggered comparable growth effects as native SY^WT^ or dsRed-SY^WT^ (data not shown). Similar as above, the strains were grown for 36h and cells were then stained with DHE and propidium iodide to determine ROS production and viability, respectively. As shown, both dyes stained cells with a dispersed cytoplasmic distribution of synphilin-1 or, occasionally, with small inclusions. In contrast, cells with larger inclusions almost completely failed to stain positive for DHE or propidium iodide (Fig. 8C,D). Again, similar observations were made with the strains combining expression of EGFP-SY^WT^ and α-Syn (data not shown). Taken together, these data favor that the large inclusions formed by SY^WT^ correspond to cytoprotective aggresomes.

**Figure 9:**
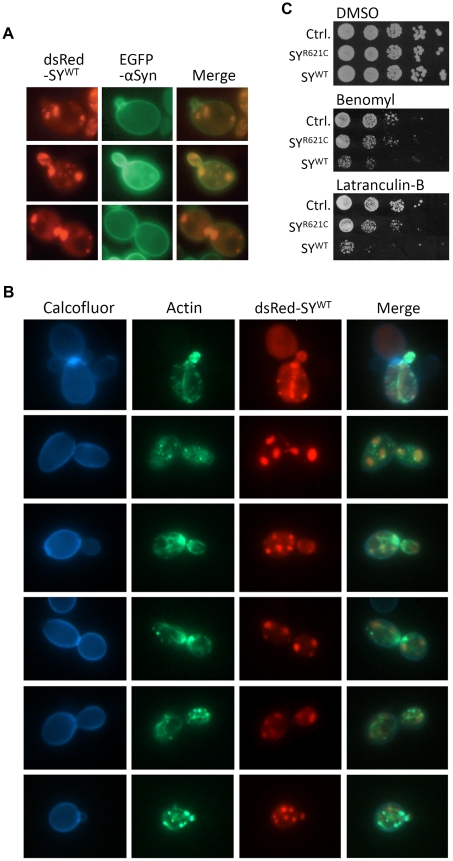
Transport of synphilin-1 inclusions along actin cables. A: Fluorescence microscopy images of late exponential *sir2Δ* cells expressing dsRed-SY^WT^ and α-Syn-EGFP showing that daughter cells inherit cytosolic synphilin-1 inclusions and plasma membrane associated α-Syn. B: Fluorescence microscopy images of late exponential wild-type cells expressing dsRed-SY^WT^ stained with Alexa Fluor 488 phalloidin to visualize actin patches and actin fibers and with Calcofluor to visualize the cell wall. Shown are the pictures obtained with the fluorescent proteins or dyes as well as the corresponding merges. C: Assessment of growth of wild-type cells with or without expression of native SY^WT^ or SY^R621C^ when plated on media supplemented with either Latranculin-B, Benomyl or the solvent DMSO.

Aggresome formation and subsequent autophagic clearance may account for the observation that, despite its higher propensity to aggregate, synphilin-1 remains less toxic than α-Syn in exponential growing yeast cells. Moreover, several studies in higher eukaryotic systems documented the essential role of sirtuins for maintenance of autophagy during conditions of nutrient starvation [58], [59]. This role of the sirtuins is most likely conserved also for yeast Sir2 [60] and, as such, it may provide a first clue for the difference in synphilin-1 instigated toxicity between ageing wild-type cells and ageing *sir2Δ* cells. Most recently, Sir2 was shown to be required for the proper CCT-chaperonin-dependent folding of actin and the establishment of cell polarity [61]. The same study then also uncovered a role of Sir2 for the efficient retention of aggregates by mother cells and the clearance of daughter cells from aggregates via polarisome-dependent retrograde transport along actin cables. During our analyses, we noticed that during mitosis the emerging buds of both wild-type and *sir2Δ* cells, indeed, inherited synphilin-1 inclusions, which were often positioned at the distal bud tip, i.e. the site of the polarisome (Fig. 9A). Interestingly, the inheritance of these inclusions occurred often before completion of chromosome segregation (see lower panels in Fig. 8A). α-Syn, on the other hand, followed a different inheritance pattern and was almost exclusively received by the growing daughter cells as plasma membrane associated protein (Fig. 9A). To demonstrate that synphilin-1 inclusions could be subject to transport along actin cables, we stained wild-type cells expressing dsRed-SY^WT^ with Alexa Fluor 488 conjugated phalloidin to visualize actin patches and filaments as well as with Calcofluor to visualize the cell wall. Although only a fraction of the cells harboring synphilin-1 stained positive for actin, our analysis not only revealed that synphilin-1 inclusions localized to actin cables but it also showed that the individual inclusions were usually flanked by an actin patch (Fig. 9B). The latter suggests that actin is recruited on the inclusions and thus that synphilin-1 inclusions trigger the delocalization of the actin cytoskeleton. To illustrate that the transport of synphilin-1 inclusions along actin cables is equally important to prevent synphilin-1 toxicity as microtubule-mediated transport and aggresome formation, we compared the growth of wild-type cells with or without expression of native SY^WT^ or SY^R621C^ when plated on media supplemented with either Latranculin-B, a drug causing actin-depolarization [61], or Benomyl, a drug preventing the polymerization of tubulin [56]. This experiment showed that synphilin-1 expression conferred hypersensitivity to both drugs and, consistent to their aggregation propensity, that for each of the drugs the effect was by far more pronounced upon expression of the wild-type protein than upon expression of the mutant (Fig. 9C).

## Discussion

During the past decade, work from several groups demonstrated the power of yeast as a model to uncover how central players in protein folding diseases incite cellular toxicity and cell death. α-Syn, for instance, was found to form inclusions at the yeast plasma membrane and the peripheral ER, to hamper protein quality control systems and to block vesicular traffic from ER to Golgi, endocytosis and vacuolar fusion [30], [31], [35], [40], [41]. Thereby, α-Syn triggers oxidative stress and mitochondrial dysfunction, which eventually leads to apoptotic and necrotic cell death [37], [38]. We now show that also the α-Syn-interacting protein synphilin-1 forms inclusions in yeast cells, which starts with the appearance of distinct foci at endomembranes and lipid droplets. These data extend the results previously obtained by *in vitro* synphilin-1 lipid binding assays and confirm the observation of co-localization of the protein with lipid droplets in transfected COS7 cells [42] and membranous structures in HEK293 cells [62]. They also corroborate the finding that synphilin-1 associates with synaptic vesicles in neurons [16].

Intriguingly, both wild-type synphilin-1 and the R621C mutant displayed much higher capacities for inclusion formation than α-Syn and we believe that this directly relates to our observation that synphilin-1 is a true lipid raft interactor in both yeast and mammalian cells, while α-Syn associates only poorly with these membrane domains, as previously shown [13], [31]. Such a correlation between lipid raft interaction and inclusion formation also holds for differences observed between wild-type and mutant synphilin-1. Indeed, consistent with data obtained in SH-SY5Y cells [24], we counted less yeast cells with inclusions upon expression of SY^R621C^ than upon expression of SY^WT^ and this is nicely recapitulated in our lipid raft binding experiments where we found that the mutant was displaced to more detergent-soluble fractions as compared to the wild-type protein. Mechanistically, this is explained by the fact that the R621C mutation reduces the lipofilicity of synphilin-1, because it is in the middle of the sequence required for phospholipid and membrane binding [42]. Furthermore, the R621C mutation is located in the second domain with ankyrin-like repeats that is critical for synphilin-1 self-assembly and the formation of deleterious aggregates [22]. We also noticed that synphilin-1 interacted with lipid rafts both as monomer and dimer, the latter being again reduced in case of the mutant protein. This led us to speculate that the interaction with lipid rafts could facilitate dimerization and further self-assembly of synphilin-1, though this certainly requires more detailed investigations. Note that synphilin-1 dimerization has been observed previously and found to involve the antiparallel binding of the central coiled-coil domain [20].

In close connection to membrane binding and inclusion formation, we found that wild-type synphilin-1 instigates a higher toxicity than the mutant protein. This is best illustrated by the observation that the expression of SY^WT^ had a more severe impact on ROS formation and viability of post-diauxic cells than the expression of SY^R621C^. In yeast, like in all other eukaryotic cells, ROS is not only generated due to mitochondrial activity but also as a consequence of sustained ER-stress and activation of the unfolded protein response (UPR) [32], [63]. Interestingly, ER-stress and the UPR were shown to enhance the biosynthesis of endomembranes as well as the formation of lipid droplets [64], [65]. Since the binding of synphilin-1 to endomembranes and lipid droplets is associated with the formation of inclusions, it is conceivable that, especially in case of wild-type synphilin-1 and certainly upon co-expression with α-Syn, cells become trapped in a vicious circle where enhanced inclusion formation triggers elevated levels of ER-stress and ROS and vice versa. Eventually, this must lead to an overflow of protein quality control systems and consequently to enhanced cell death, the latter being confirmed by our data.

The observation that expression of SY^WT^ is more cytotoxic than expression of SY^R621C^ in yeast disagrees with the initial reports that the R621C mutation would be specifically associated to PD [24]. It also seems to contradict the data suggesting that particularly the expression of the R621C mutant rendered HEK293 cells more susceptible for staurosporine-induced cell death [24]. However, this effect is only observed when the HEK293 cells were treated with proteasome inhibitors and therefore it may be due to a more pleiotropic pharmacological effect rather than to properties associated to the mutant proteins itself under normal physiological conditions.

Synphilin-1 was initially characterized as α-Syn interacting protein based on a yeast two-hybrid screen [15], indicating that both proteins maintain their interaction properties when expressed in *S. cerevisiae*. Our data confirmed the co-localization of SY^WT^ or SY^R621C^ and α-Syn in inclusions and established that both SY^WT^ and SY^R621C^ do enhance α-Syn inclusion formation also in yeast, similar as previously shown in mammalian cells [15], [19], [20]. This stimulatory effect on inclusion formation is apparently not mutual, because the expression of α-Syn had only a minor impact on inclusion formation by synphilin-1. An explanation for this observation is straightforward. Indeed, several studies established that aggregation of α-Syn in mammalian and yeast cells requires the interaction with membranes via its N-terminus [1], [9], [10], and the same sequence was shown to be essential for the interaction with the coiled-coil domain of synphilin-1 [20], [66]. Thus, when α-Syn binds to membranes and is engaged to form aggregation nuclei, the sequence needed for interaction with synphilin-1 is occupied and no longer available. Conversely, the coiled-coil domain of synphilin-1 does not overlap with the phospholipid binding site [42] and thus remains exposed for recruitment of α-Syn even when synphilin-1 is bound to membranes as a dimer [20].

The enhanced inclusion formation by α-Syn upon co-expression of SY^WT^ or SY^R621C^ was paralleled by an increased level of its phosphorylation at S129. As reported previously, and similarly to what was described in mammalian cells, S129 phosphorylation of α-Syn in yeast cells can be mediated by different casein kinases, of which the two plasma membrane resident kinases seem to determine α-Syn-mediated toxicity [31]. Although it is still a matter of debate [67], numerous studies performed in cellular and transgenic models as well as on human brain suggested that phosphorylation at S129 is specifically associated with α-Syn neurotoxicity and therefore represents an early marker for PD [13], [46], [68], [69], [70], [71]. We observed that only the combined expression of α-Syn with SY^WT^ triggered a more severe growth defect when yeast cells were actively dividing, while both the combined expression of α-Syn with SY^WT^ or with SY^R621C^ resulted in enhanced toxicity when cells were kept in stationary phase for further ageing. This suggests that the relationship between S129 phosphorylation and toxicity of α-Syn is growth-phase dependent and that it may only be relevant in post-mitotic cells. Noteworthy, it has been reported that the phosphorylation of α-Syn at S129 is not critical for its interaction with synphilin-1 [72]. This could explain why the increase in S129 phosphorylation observed in our experiments upon co-expression of synphilin-1 is less pronounced than the increase in α-Syn inclusions.

Another striking observation is that the expression of SY^WT^ or SY^R621C^ was less toxic for exponential yeast cells than the expression of α-Syn, although both proteins displayed much higher capacities to form inclusions. This underscore that there is no correlation between the level of cytotoxicity and the absolute number of inclusions being formed in yeast cells, which is consistent to previously published reports [10], [31], [32]. In fact, studies in mammalian cell lines and transgenic mice indicated that synphilin-1 can form deleterious aggregates as well as cytoprotective aggresomes [19], [22], [23]. Interestingly, these properties are associated with distinct protein domains, i.e. the aggregation of synphilin-1 is dependent on the coiled-coil domain and the second ankyrin-like repeats domain, while aggresome formation requires the first ankyrin-like repeats domain [22]. Aggresomes are larger than aggregates and they are formed near the centrosome by microtubule-dependent transport of aggregated proteins in response to a failing proteasome. Their formation is usually considered to be cytoprotective response because aggresomes can be cleared via autophagy, thereby providing the cell an additional mechanism to deal with misfolded and insoluble proteins. We observed that smaller synphilin-1 inclusions transformed into one or a few larger inclusions located near the nucleus when the cells traversed the diauxic shift to enter stationary phase. Furthermore, cells harboring such large inclusions failed to stain positive with DHE and propidium iodide. These observations suggest that both wild-type and mutant synphilin-1 are subject to aggresome formation in yeast as well. This may explain, at least in part, why the aggregation of synphilin-1 does not translate into an extensive growth defect. Furthermore, the formation of synphilin-1 aggresomes may also lead to sequestration of noxious forms of α-Syn, thereby providing an alternative to deal with this protein as well, on condition that the overall capacity for aggresome formation is not exceeded. Apparently, this is still the case for combined expression of SY^R621C^ and α-Syn in late exponential cells. Note, however, that the cytoprotective function of aggresomes has been questioned since their occurrence is often associated with disease states where they are believed to trigger deregulation of normal cellular homeostasis [73]. In our yeast model it is obvious that aggresome formation did not prevent that SY^WT^ and SY^R621C^ incited cytotoxicity during ageing, certainly not when their expression was combined with that of α-Syn. One may argue that aged yeast cells are more vulnerable to heterologous expression of synphilin-1 and α-Syn because their mechanisms to handle misfolded proteins are already operating at maximal capacity due to elevated levels of damaged endogenous proteins. In addition, the overwhelming aggregation of synphilin-1 and excessive aggresome build-up during growth could trigger the deregulation of autophagic processes when these become activated in response to nutrient starvation [74]. For instance, the autophagic clearance of aggresomes could interfere with the quality control and recycling of essential cellular constituents, such as mitochondria by means of mitophagy, as these processes are all dependent on a common core machinery [75]. Consistent with the latter scenario is the observation that, despite its extensive aggregation and formation of large inclusions, synphilin-1 did not trigger additional cytotoxicity when expressed in cells lacking the sirtuin Sir2, a protein required to maintain the autophagic flux upon nutrient deprivation [60].

The finding that Sir2 is an essential mediator of synphilin-1 toxicity also pointed us to another process that appears to be influenced by the presence of aggregates and aggresomes and that is particularly relevant during mitotic growth. Indeed, a recent study identified Sir2 as being essential for the proper CCT-chaperonin-dependent folding of actin and the establishment of cell polarity [61]. Sir2 thereby controls the efficient retention of aggregates by mother cells and the clearance of aggregates from daughter cells via an actin dependent retrograde transport. Careful examination of wild-type cells revealed that synphilin-1 inclusions localized to actin cables and that the individual inclusions were usually flanked by an actin patch. These observations suggest that synphilin-1 inclusions trigger the delocalization of the actin cytoskeleton as to allow transport of aggregates along actin cables. Furthermore, we found that the expression of SY^WT^ in wild-type cells caused hypersensitivity to Latranculin-B, an actin-depolarizing drug. It is therefore tempting to speculate that this actin-mediated transport of synphilin-1 inclusions permits to produce a more long-lived progeny and that it contributes to the formation of aggresomes in mother cells. However, these issues remain to be investigated in more detail in future studies.

## Materials and Methods

### Cloning of Synhilin-1 and α-synuclein

The synphilin-1 cDNA was isolated from a hippocampal cDNA library via PCR amplification using the primers CATGCCATGGAAGCCCCTGAATACC and CCGCTCGAGTTATGCTGCCTTATTCTTTCC that included, respectively, a NcoI and XhoI restriction site for cloning into the pYX212 plasmid, which allows expression from the constitutive *TPI1* promoter. The sequence for dsRed was amplified with the primers CATGCCATGGATGGACAACACCGAGGACG and CATGCCATGGCTGGGAGCCGGAGTG both including a NcoI site for in-frame cloning into the pYX212-synphilin-1 plasmid constructed above. The N-terminally tagged EGFP-synphilin-1 plasmid was made using the gateway technology, where the expression cassette was placed under control of the glyceraldehydes-3-phosphate dyhydrogenase (*TDH3*) promoter. The cDNA of the clinical synphilin-1 R621C mutant was a gift from Dr. R. Krüger (Center of Neurology, Tübingen, Germany) and was also subcloned into the pYX212 plasmid using the primers described above, allowing to express the protein as native or dsRed-tagged version. Generation of the pUG23/α-synuclein and Yep181/α-synuclein vectors has been described previously [41]. The plasmids used to transfect Human H4 neuroglioma cells were described previously [76], [77].

### Yeast cultures and determination of cell survival

In this study we used the wild-type BY4741 (*MATa his3Δ leu2Δ met15Δ ura3Δ*) strain. The Gietz method was used for transformations [78]. All strains were grown on SC medium containing 0.17% yeast nitrogen base (Difco, Lawrence KS, USA), 0.5% (NH_4_)_2_SO_4_, 30 mg/l adenine, 80 mg/l histidine, 30 mg/l of all other amino acids (leucine and/or the pyrimidine uracil were omitted to serve as selection marker) and 2% glucose (SCD). In order to test growth retardation caused by native α-Syn or synphilin-1, alone or in combination, the growth profiles of the corresponding strains were compared to that of a control strain transformed with the empty vectors. Overnight precultures of at least three independent transformants were used to inoculate new cultures in SCD medium at a starting OD_600_ of 0.01 in microtiter plates, which were incubated at 30°C without shaking. The growth profiles were then established by measuring OD_600_ in a DTX880 multimode detector (Beckman Coulter, California, USA) until the stationary phase was reached. For growth assays on solid medium, a serial dilution of exponentially growing cells was made in SCD medium ranging from an OD_600_ of 1 to 0.0001. Of these dilutions, 5 µl was spotted on SCD agar plates, which were then incubated at 30°C for at least 48 h. To determine survival upon α-synuclein and synphilin-1 expression, cells from overnight cultures were inoculated in tubes on SCD to OD_600_ 0.1, and grown at 30°C and 145 rpm. At different times after inoculation, cultures were subjected to cell counting using a CASY cell counting device (Schärfe system). 500 cells were plated on YPD agar plates, and colony forming units were determined after 2 days with a Microbiology colony counter (LemnaTec GmbH, Würselen, Germany) and processed using SAWmicrobio version 3.1. For chronological ageing experiments, cultures were grown in flasks with vigorous shaking and processed as previously described [37], [79]. Notably, at least three independent transformants were tested for the survival plating to rule out clonogenic variation of the effects.

### Cell cultures

Human H4 neuroglioma cells (HTB-148; ATCC, Manassas, VA, USA) were maintained in OPTI-MEM (Gibco/Invitrogen corporation, Carlsbad, CA, USA) supplemented with 10% fetal bovine serum. H4 cells were passaged 24 hours prior to transfection and plated in 10 cm dishes for western blot analysis. Cells were transfected with equimolar ratios of plasmids using Fugene6 (Roche, Mannheim, Germany) according to the manufacturer's instructions. To prepare primary neuronal cultures, cortices from 16 days old embryos were dissected and conserved in L15 medium (Gibco/Invitrogen corporation, Carlsbad, CA, USA) supplemented with 30 mM glucose till dissociation. Mechanical dissociation of the mixed glial cells was performed using flamed Pasteur pipets and the cells were plated at a density of 200000 cells/cm^2^ in poly-D-lysine and laminin-coated plates. For dissociation, plating and maintenance, we used Neurobasal medium supplemented with 200 mM glutamine, 2% B27 supplement and 1% antibiotic/antimycotic agents (Gibco/Invitrogen corporation, Carlsbad, CA, USA).

### Western blot analysis

Yeast samples for western blot analysis were prepared according to Zabrocki and co-authors [41]. For quantitative analysis, strains were grown to mid-exponential phase (OD_600_ of 2.0) and an equal amount of cells was taken for protein extraction. Proteins were then separated by SDS-PAGE. Human H4 neuroglioma cells were washed with cold phosphate buffered saline (PBS) 36 hours post transfection cells, and lysed with NP-40 buffer in the presence of protease inhibitor cocktail (Roche, Mannheim, Germany). Lysates were cleared from debris by a 8000xg centrifugation for 15 min at 4°C and were then subjected to SDS-PAGE. After blotting, the filters were washed three times in TBS with 0.1% Tween (TBS-T, pH 7.4), and bands were stained using antibodies listed below, followed by incubation with HRP labeled secondary antibodies. After washing three times in TBS-T, the immunoblots were developed using ECL. The primary antibodies used were specific for Pma1 (kind gift from Dr. B. André, ULB, Belgium), synphilin-1 (Sigma Aldrich, St. Louis, MO, USA), α-synuclein (Sigma Aldrich, St. Louis, MO, USA and BD Transduction Laboratories, San Jose, CA, USA), phospho-serine 129 (P-S129) α-synuclein (Wako Chemicals USA, Inc., Richmond VA, USA and Abcam, Cambridge, UK) and flotillin-1 (Cell Signalling, Danvers MA, USA). To quantify expression of wild-type and mutant synphilin-1 in the yeast strains, the endogenous alcohol dehydrogenase Adh2 served as internal standard.

### Microscopy and determination of cell death markers

Fluorescence microscopy of yeast was performed with a Leica DM4000B microscope. Transformants with the expression cassettes for EGFP- or dsRed-fused α-Syn or Synphilin-1 were inoculated in tubes on SCD to OD_600_ 0.1 and incubated for 16 hours at 30°C as described previously [41]. The proportion of cells with the α-synuclein and synphilin-1 inclusions within the population was then determined by manual inspection of at least 600 cells per strain. Data from different experiments were combined to calculate the significance of the data using a t-test.

Yeast intracellular lipid droplets were stained with the non-polar BODIPY 505/515 fluorescent probe. The fatty acid C1-BODIPY 500/510-C12, which serves as fluorescent precursor for phospholipid synthesis, was used to stain lipid particles. Both stainings were done with the same protocol. Briefly, cells were grown on selective medium till OD_600_ of 0.4 at 30°C. Then the fluorophore was added to an end concentration of 5 µM and the cells were further incubated for 1 h. After centrifugation, the cells were washed three times in synthetic medium, resuspended in medium and visualized under the microscope. For filipin staining of sterols the cells were concentrated by a brief centrifugation and filipin (Sigma Aldrich, St. Louis, MO, USA), dissolved in dimethyl sulfoxide, was then added at a final concentration of 5 µg/ml. Staining with DAPI, Calcofluor or Alexa Fluor 488 conjugated phalloidin (Molecular Probes, Eugene, OR, USA) involved the fixation of cells with formaldehyde as previously described [80].

Tests for apoptotic and necrotic markers (using AnnexinV/PI co-staining) as well as ROS-accumulation using the superoxide-driven conversion of non-fluorescent dihydroethidium (DHE) to fluorescent ethidium were performed and quantified using flow cytometry as described previously [37]. Significance of the data was determined by t-test analysis.

Transfected H4 cells were washed with PBS and fixed with 4% paraformaldehyde for 10 min at RT. After washing with PBS cells were permeabilized in TBS containing 0.1% Triton X-100 for 20 min at RT. After blocking in 1.5% normal goat serum containing TBS for 1 hour cells were incubated with primary antibody for 2 hours at RT or overnight at 4°C (mouse anti-Syn-1; BD Transduction Laboratories, San Jose, CA, USA) followed by washing with PBS and secondary antibody incubation for 1 hour (goat anti-rat IgG-Alexa488, 1∶300; Molecular Probes, Eugene, OR, USA). After a final wash, slides were mounted with aqueous mounting solution (GVA; Zymed, San Francisco, CA, USA) and subjected to fluorescence microscopy. The proportion of cells with the α-synuclein inclusions within the population was then determined by counting. All data were analyzed using a t-test.

Primary neuronal cultures (11 DIV) from embryonic mouse cortex were analyzed by confocal microscopy. The cells were incubated with Alexa Fluor 488 conjugated cholera toxin subunit B (CT-B; Molecular Probes, Eugene, OR, USA), which serves a marker for lipid rafts [45], at a concentration of 10 µg/ml in D-PBS for 1 h on ice. Cells were washed with D-PBS and fixed in cold 4% paraformaldehyde in 0,1 M phosphate buffer, pH 7,4, for 30 min at room temperature. Permeabilization was carried out in 0.2% Triton X-100 in phosphate-buffered saline for 10 min. After a 30 min saturation in 2% bovine serum albumin, immunostaining was carried out using goat anti-synphilin 1 (AHP 593; Serotec, Oxford, UK). Synphilin-1 staining was detected with a donkey anti-goat IgG (H+L) antibody coupled to Alexa Fluor 568 (Molecular Probes, Eugene, OR, USA). Cells were mounted with the Vectashield mounting medium with DAPI (Molecular Probes, Eugene, OR, USA). Slides were analyzed with a Zeiss LSM710 confocal laser scanning microscope. A Zeiss LSM710 confocal laser program was used to visualize co-localization between synphilin-1 and Alexa Fluor 488®conjugated CT-B.

### Isolation of detergent-resistant lipid raft fractions

For yeast, an amount of exponentially grown cells equivalent to an OD_600_ of 20 was taken to perform the isolation of lipid raft fractions. The exponential cells were washed once with chilled water and lysed using a standard protocol with glass beads. Lysis was performed in TNE buffer (50 mM Tris–HCl, pH 7.4, 150 mM NaCl and 5 mM EDTA) with addition of inhibitors (1 µM benzamidine, 1 µM PMSF and a cocktail of protease inhibitors (Roche, Mannheim, Germany)). Lysates were centrifuged at 500 g for 5 min at 4°C. 0.2% Triton-X-100 was added to the lysates and these were then incubated on ice for 30 min. Fractions were mixed with OptiPrep (Sigma Aldrich, St. Louis, MO, USA) solution to reach 35% concentration (v/v), then overlaid with 1.2 ml of 30% OptiPrep solution (v/v) and 200 µl of TNE. The samples were centrifuged at 259,000 g for 16 h at 4°C in SW55Ti rotor (Beckman Coulter, Brea, CA, USA). Nine equal fractions were taken and precipitated by adding 50% trichloroacetic acid. Samples were centrifuged and protein pellets were resuspended in 2×SDS-PAGE buffer. Equal volumes of particular fractions were analyzed by SDS-PAGE and western blotting. For lipid raft analysis of mammalian cells, 11 DIV neuronal cultures were washed with PBS and harvested immediately in TBS containing 1% triton-X-100, phosphatase inhibitors (1 mM Na_3_VO_4_, 0.1 µg/ml okadaic acid) and a protease inhibitors mix (Roche, Mannheim, Germany) and then incubated on ice for 30 min. Lysates were centrifuged at 1,000 g and supernatants were collected. The total protein concentrations were determined by using the BCA protein assay kit (Pierce Biotechnology, Rockford, IL, USA). A total amount of 1 mg of protein extracts was used to perform the isolation of lipid raft fractions. The lysates were mixed with a sucrose solution to reach 40% concentration (m/v) and then overlaid with 6 ml of 35% sucrose (m/v) and subsequently with 1.3 ml of 5% sucrose (m/v). The samples were centrifuged at 200,000 g for 18 h at 4°C in SW60 rotor (Beckman Coulter, Brea, CA, USA). Eight equal fractions were taken and every fraction was precipitated by adding 100% trichloroacetic acid. Samples were centrifuged and protein pellets were resuspended in 2×SDS-PAGE buffer. Equal volumes of particular fractions were analyzed by SDS-PAGE and western blotting.
